# Assessment and Improvement of Masticatory Performance in Frail Older People: A Narrative Review

**DOI:** 10.3390/jcm12113760

**Published:** 2023-05-30

**Authors:** Martin Schimmel, Noemi Anliker, Gabriela Panca Sabatini, Marcella Silva De Paula, Adrian Roman Weber, Pedro Molinero-Mourelle

**Affiliations:** 1 Department of Reconstructive Dentistry and Gerodontology, School of Dental Medicine, University of Bern, 3010 Bern, Switzerland; 2 Division of Gerodontology and Removable Prosthodontics, University Clinics of Dental Medicine, University of Geneva, 1211 Geneva, Switzerland; 3 Department of Prosthodontics, University of São Paulo (USP), São Paulo 05508-900, Brazil; 4 Department of Prevention and Oral Rehabilitation, Universidade Federal de Goiás, Goiania 74690-900, Brazil

**Keywords:** geriatrics, frail elderly, frailty, oral health, tooth loss, dental care for age

## Abstract

According to the World Health Organization (WHO), the estimated number of older adults is around 962 million and is projected to increase to 2.1 billion by 2050. The oral frailty concept is associated with gradual oral function loss in relation to aging. There is a need to emphasize the improvement of oral function based on an evaluation of masticatory performance in patients with various oral conditions or systemic diseases and especially in the frail elderly. The present narrative review presents an overview of the current state of the assessment and improvement of masticatory performance in frail older people. To fully encompass oral frailty, oro-facial hypofunction, or oro-facial fitness, dental Patient Reported Outcomes (dPROs) should be included; nevertheless, there are limited evidence-based rehabilitation approaches. The concept of oral frailty, oro-facial hypofunction, or oro-facial fitness should involve dental Patient Reported Outcomes (dPROs), and in this sense, there are only a few evidence-based rehabilitation procedures to improve oro-facial hypofunction besides prosthodontics. It must be considered that reduced neuroplastic capacity in old individuals might preclude a positive outcome of these strategies that might need to be accompanied by functional training and nutritional counseling.

## 1. Introduction

According to the World Health Organization (WHO), as of 2020, the number of older adults (defined as people aged 60 years and older) worldwide is estimated to be around 962 million. This number is projected to increase to 2.1 billion by 2050, which represents a significant demographic shift toward an aging population [1].

This shift is related to an increased life expectancy and a reduced birth rate, as observed primarily in the Western world, but the same effect is present in developing countries. Nowadays, the so-called baby boomer generation has arrived at medical and dental practices, and this trend will be progressing during the next ten to twenty years [2]. Due to the current social aspects, especially in industrialized countries, these individuals have high expectations in relation to their oral health and orofacial function. If teeth need replacement, many of them expect stable, functional, esthetic and predictable tooth replacements. In this sense, this group of patients is little inclined to make compromises regarding their social life and food choice and often demonstrate a high willingness to invest in “high technology” treatment and prostheses [3].

Aging is a continuous process, and during this period, individuals will be subjected to progressive degenerative changes in their state of mental, cognitive, and general health [4]. In the last period of life, during old age, individuals might become frail or finally dependent on care. Laslett [5] introduced the term third age, which compromises a phase of retirement with social integration, high levels of activity and often very active baby boomers. Later, the term “fourth age” was introduced, which is characterized by a unique excess of women over men, higher levels of comorbidity and institutionalization, and greater consumption of medical and care services [6]. In a specific description, “third age” exemplifies positive characteristics, and “fourth age” dysfunction and death on biological, functional, or quality of life characteristics [7].

Considering this current epidemiological trend, aging is related to frailty, which is defined as “a state of vulnerability to poor resolution of homeostasis following a stress and is a consequence of a cumulative decline in multiple physiological systems over a lifespan”. Often, individuals will expect a gradual decline of the physiological health reserve; nevertheless, the frailty state includes multiple interrelated physiological system disorders that will decline in a more accelerated way with the consequent initiation of the failure of homeostatic mechanisms [8].

With respect to the frailty state, it is important to remark that complex aging mechanisms are involved in this state, which will promote a continuous cumulative decline in the physiological systems that will weaken the homeostasis of these individuals, making them more vulnerable to the consequent changes in the state of health [9]. For dental medicine, oral frailty is a relatively new concept that can be associated with gradual oral function loss in relation to aging [10]. This state is characterized by the presence of whole limitations and deficiencies that produce a rapid deterioration in daily oral functions, such as tooth loss, poor oral hygiene, the existence of insufficient dental prostheses and/or difficulty chewing associated with age-related swallowing changes [8].

Tooth loss plays a significant role in the oral health and quality of life of frail older people since this condition triggers a deterioration of all masticatory and swallowing functions [11]. In the Western world, this condition has been decreasing in the last 20 years due to the improvements in oral health prevention and care, especially for edentulism [12]. Nevertheless, it is still highly prevalent in frail and dependent older adults. Edentulism increases exponentially with age; it is often associated with multimorbidity and is highly dependent on socio-economics factors [13]. In addition to tooth loss, oral health challenges in the old population include dental caries, periodontal disease, dry mouth, oral precancer/cancer, denture-related conditions, masticatory impairment, dysphagia and aspiration pneumonia [14,15]. The consequent oral and dental care of this patient should be: ensuring access to dental care for the elderly, establishing an infection- and pain-free oral cavity, long-term prevention of oral infections, fostering oral health-related quality of life factors (such as keeping 20 teeth or more and avoidance of removable dental prostheses), the maintenance or improving masticatory function and ensuring acceptable oral aesthetic appearance and the maintenance of patient autonomy [16]. The needs for care within the frail aged population are undergoing rapid changes as the baby boomer generation reaches retirement age. Traditional top-down healthcare decision-making that relies on passive acceptance of offered treatments is no longer sufficient. Normative and expressed needs may diverge, and patients are increasingly participating in research to inform best practices. Thus, there is a growing need for more collaborative, partnership-based decision-making with the patient to ensure that care aligns with their needs and preferences [17].

Considering the previous statements, there is a need to emphasize the improvement of oral function based on an evaluation of masticatory performance in patients with various oral conditions or systemic diseases and especially in the frail elderly. The aim of the present narrative review is to present a concise but comprehensive overview of the current state of the assessment and improvement of masticatory performance in frail older people.

## 2. A Conceptual Model of Oro-Facial Health with an Emphasis on Function

Historically, trends in dental medicine have often led to an artificial separation in dental education, research, patient care and public health policy from general medicine and its disciplines [18]. This obvious limitation is now changing and evolving, and therefore current educational trends will allow current and future clinicians to consider the orofacial system in a broader context of health and function. In relation to these changes, in the last decade, both in undergraduate and postgraduate dental education and in daily clinical practice, the domains of quality of life and patient-centered values have gained importance with an emphasis on oro-facial function [19].

In 2014, the Meikirch model was developed with a new dynamic definition of the health concept [20]. The definition was not only based on static immunological systems state but health results throughout the life course when individuals’ potentials—and social and environmental determinants—suffice to respond satisfactorily to the demands of life. During life, the biologically given potential decreases as a result of the general, irreversible process of aging. Parallel to this decline and compensating for the possible negative implications on the orofacial functional capacity, the personally acquired potential increases, ensuring the state of health [21]. Most oro-facial conditions, such as periodontitis, tooth loss, impaired oral food processing or hyposalivation, are chronic and hence require attention for prolonged periods of time. Conditions of the orofacial system might be the origin or even can modify general diseases such as aspiration pneumonia, or general conditions such as cancer and the treatment thereof might manifest themselves in the oral cavity and hamper oro-facial function [15,22]. Nowadays, a common view is that health care should provide a cure, whereas providing long-term care and maintaining the quality of life (QoL) to an acceptable standard, from a person’s point of view, has gained little attention so far [17,23,24].

The oral health-related quality of life (OHRQoL) is a concept that is based on the idea of patient-based medicine and is a useful tool that clinicians and researchers can use to understand or assess the oral state, dental treatment or a related condition [25,26]. OHRQoL includes biological, social, and psychological aspects and is gaining importance in the context of oro-facial frailty and oro-facial hypofunction. It should be considered that oral function, oro-facial pain, oro-facial appearance and psychosocial impact are related to the reasons why patients seek help from dental and medical professionals [27,28].

As part of the conceptual health, to achieve favorable treatment outcomes, researchers and clinicians measure the different states that are involved in dental medicine, such as the biological status, the clinical status and the patient functional status (chewing function, oral diadochokinesis) [29]. Based on this state, specific dental Patient-Reported Outcomes (dPROs) have been developed to measure the influence of oral health on aspects of daily living, patient satisfaction with dental care, oral health and treatment outcome and patients’ self-perception of oral health, dental treatment or esthetics [29]. This state can be measured by means of questionnaires (instruments) or by a qualitative approach that is oriented to reflect the multidimensional model of OHRQoL. These methodologies have been widely investigated and validated and currently are one of the most used and useful tools for assessing oral health status. Among others, the most used in gerodontology are a denture satisfaction index (DSI); visual analog scales (VAS), an oral health impact profile (OHIP) and the Geriatric Oral Health Assessment Index (GOHAI) [30,31,32,33].

The use of these questionnaires can give a baseline assessment and, therefore, valuable information and diagnosis to develop a proper treatment plan to improve the masticatory ability, especially in such patients that are dependent or impaired [34]. Considering the conceptual model of oro-facial health and considering the limitations of frail ancient populations, some physiological requirements are in relation to oral function. It should be noted that oral function not only includes eating, swallowing or speaking ability as physical aspects but also has psychosocial and environmental aspects.

The condition of being fit in terms of oro-facial health, commonly referred to as the vitality of the oro-facial system, involves the lack of effective management of physical and mental ailments, pain, and negative environmental or social aspects. This enables individuals to meet the demands of everyday life while also facilitating natural oro-facial functions such as sensation, taste, touch, bite, mastication, deglutition, articulation, yawning, singing, kissing and different countenances. Comorbid diseases such as frailty may negatively affect the oro-facial functional capacity and may result in dysfunction and disease. It was described that an association between the presence of poor oral health status is associated with polypharmacy and multimorbidity, and therefore, orofacial fitness will be affected [35]. Although the interest in this topic has been growing, there is still a lack of widespread, validated, easy-to-use instruments that help to distinguish between states of orofacial fitness as opposed to orofacial hypofunction [24].

## 3. Factors Influencing Mastication

Mastication is one of the most important physiological functions since it is interrelated with eating and swallowing depends on it [36]. It is well documented how the aging process is related to a deterioration of the masticatory function involving the decrease of occlusal forces and the motor function of the tongue and peri-oral muscles [37]. The main consequence of functional progressive aging will be an impairment of the masticatory efficiency, food bolus formation and swallowing, and therefore digestion and nutrient absorption will be compromised, leading to insufficient nutrition [37,38]. As with any other body function, masticatory function is a multifactorial process that involves oro-facial structures and function, individuals’ physiological aspects, the environment and general health [24].

Masticatory function depends not only on tooth- and prosthesis-related factors. Edentulous adults have usually already reached an advanced age and thus frequently exhibit age-related comorbidities [39].

Increasing evidence also suggests that not only impaired cognitive function leads to oral dysfunction but indeed, masticatory difficulties could be a causal contributor to the onset of neurodegenerative diseases such as dementia [40,41,42,43,44,45]. The favorable masticatory function could therefore be a protecting factor in terms of increased cerebral blood flow in patients that already suffer from neurodegenerative conditions [46].

With increasing disease, saliva-inhibiting medications are also taken more frequently, which can lead to a variety of problems. The lack of saliva causes poorly retained removable dentures and often pain due to the lack of the mucosa protective effect of saliva. In addition, food cannot be lubricated, which greatly complicates the shaping and oral/esophageal transport of the food bolus. Thus, chewing efficiency is also significantly dependent on saliva quantity and consistency [47].

Also, the influence of the tongue, palate, cheek, and lip on chewing function should not be underestimated. As food particles are crushed between the chewing surfaces, these structures shape the bolus and reposition it between the dentition between masticatory cycles [48,49]. For example, stroke patients whose innervation, strength, and mobility of these structures are impaired also show decreased masticatory efficiency [50]. The decline in muscular coordination ability may be a physiological sign of aging, as it is with handwriting, but its effect on chewing function is poorly documented [51]. In contrast, a decline in chewing ability has been demonstrated in patients with neurodegenerative diseases [52]. In the advanced stage of Alzheimer’s dementia, the brain no longer knows how to generate chewing and swallowing movements, and even when food is placed in the patient’s mouth, feeding difficulty occurs in older adults with dementia [53,54].

Another aspect related to masticatory function is the age-related atrophy of the large jaw sphincters, which is further accelerated by edentulism [55]. Newton et al. have shown that overdentures supported by natural roots counteract the atrophy of jaw elevators [56]. To date, there is limited evidence, but it appears that implant-supported/retained dentures may also inhibit this atrophy [57]. This underscores the preventive benefits of implant-supported restorations in the edentulous patient.

Although the masticatory function of edentulous patients can be significantly increased by stabilizing, especially the lower denture, this does not automatically have a positive influence on diet or nutritional status [24]. Nutrition of the elderly depends on many factors, such as limited mobility, appetite, budget, depression and long-established habits [58]. Therefore, about up to one-third of the old living at home show malnutrition or undernutrition, and the proportion is likely to be even higher among institutionalized seniors [59,60]. In a Geneva study, it was shown that in a population sample of people over 80, 40% had less than three foods or spoiled foods, and 10% had no food at all in the refrigerator [61].

Chewing as a physiological function includes food-related aspects such as appetite, expectation, smell, taste, texture, temperature, preferences and conditioning. As previously mentioned, the multifactorial of the masticatory function not only involves the oral function but co-factors of oral food processing, such as the vision, the smell, the taste, the rheological properties, individuals’ expectations and the cultural/religious context.

## 4. Assessment of the Masticatory Performance in Frail Older People

It is evident how individual progressive aging will produce a series of changes at the oro-facial level that will be aggravated by tooth loss. Setting aside the classic extraoral physical signs and the intraoral state that characterize the edentulous state, the most critical change is chewing impairment [11,62,63]. If this situation is considered, it is of paramount importance for the clinician to be able to assess this state of mastication.

Numerous methodological procedures have been used to describe the masticatory process, but there is occasional overlap in the terminology used to describe these techniques. It is crucial to standardize these terms to enable comparisons across studies. Care should especially be taken to distinguish between the terms “chewing efficiency/performance” for an objective clinical evaluation of the chewing function and “masticatory ability”, which comprises the subjective evaluation by the individual [64].

Therefore, the objective evaluation of the mastication will be based on the individual’s chewing efficiency or chewing performance, which may be assessed using well-validated tests. Furthermore, it was previously mentioned within the scope of the current oral health concept that there is a need to also use an evaluation from the individual’s point of view to further assess compensation mechanisms. Due to alterations in the orofacial systems, as individuals age and become frail, their chewing behavior tends to change. Peyron et al. have shown that for every year of life, there is an average increase of 0.3 cycles per sequence, which refers to the number of chewing cycles performed before swallowing [65]. Additionally, there is a gradual increase in the mean summed EMG (electromyographic) activity per sequence, which is a measure of the muscle activity involved in chewing. Furthermore, older individuals also tend to exhibit changes in cycle and opening duration at the beginning of the chewing sequence. Specifically, these durations decrease with age, which suggests that there may be alterations in the coordination of chewing movements as individuals get older [65]. Considering these statements, the best way to evaluate mastication will be based on a combination of patient-based and laboratory-based methods [36].

Hence, from a clinical point of view, the sole evaluation of chewing efficiency/performance is not sufficient to comprehend the old and/or frail patients‘ adaptive or maladaptive behavior in relation to mastication or bolus preparation.

## 5. Chewing Efficiency

Objective evaluations might comprise the use assessment with “breakable” foodstuff or test food such as nuts or silicone cubes (Optocal), plastic or elastic test foods including chewing gum or wax, or finally, shearable specimens including gummy jelly of various hardness.

The glossary of prosthodontic terms defines chewing or masticatory efficiency as the “degree of effort needed to grind food to a standardized level of comminution” [66] and has been assessed by several methods, as shown in Table 1. One important term is median particle size, which is used to describe the food bolus. When investigating how crumbly foods such as nuts break down during chewing until they form a food bolus consisting of small particles, this bolus can be studied. When analyzing the distribution of particle size using techniques such as optical scanning or sieving, this can be quantified in terms of the median particle size. By chewing the food a specific number of times, the particle size distribution can be used to evaluate masticatory or chewing performance [64]. As an objective test to analyze particle size distribution, the empirical equation of the Rosin-Rammler formula can be used, which is defined by two parameters—the median particle size (D50) and the cumulative distribution of particle sizes in a sample [67]. A further example is the “carrot test”; carrots as test food were applied in research to define a minimum masticatory efficacy of healthy individuals to be classified as such with regard to chewing function. Carrots were also used in geriatric research to screen for deficient masticatory function with a simpler evaluation based on the visual evaluation of chewed carrots. Tests assessing chewing efficiency with these breakable test foods might still be the gold standard of assessing masticatory function due to their long history and widespread application in research; however, they are expensive and laborious to use due to the need for laboratory-based analysis and furthermore, might not be safe in frail elders who might suffer from dysphagia.

Another objective to describe chewing efficiency is shearing tests. Elastic “shearable” foodstuff refers to foods that have a certain level of elasticity. This property is often associated with certain types of meat, such as beef or pork, as well as some plant-based foods, including mushrooms. The amount of deformation that occurs before the food breaks depends on the food’s elasticity and other properties, such as its moisture content and texture. Considering these different aspects, researchers at the University of Auvergne developed a model food material that is designed to simulate the mechanical properties of natural foods, such as hardness, elasticity, and texture. It is a standardized, non-nutritive material made from a mixture of synthetic polymers, invented by the University of Auvergne, faculty of dental surgery in France. Likewise, the Glucosensor© test (GC, Lucerne, Switzerland) is a technique that involves crushing a jelly within a 20-s time frame and dissolving it in water to determine the quantity of glucose (mg/dL) released. A specimen is deemed to have inferior masticatory function when the glucose concentration measured is less than 100 mg/dL [37]. This test is widely applied in Japan and was adapted for the diagnoses of oral hypofunction by the Japanese Society of Gerodontology [10].

In terms of texture, a plastic “deformable” foodstuff is a food material that can be easily molded or reshaped without breaking or cracking. In order to facilitate the objective measurement of chewing function, color mixing tests have been developed. Two-color test foods (i.e., wax, chewing gum, colored gelatin) are used [68,69,70,71]. The degree of color mixing achieved and the shape of the resulting bolus obtained after a given number of chewing cycles can be used as a measure of chewing efficiency. The two-color mixing test correlates significantly with the “sieve method” and is particularly suitable for subjects with reduced chewing function [72].

Own investigations [70,73] could show that the color mixing degree of a two-color chewing gum can be approximately described by a logarithmic function (log10) with the base “number of chewing cycles”. Here, the test subject is offered conventional, commercially available chewing gum in the colors blue and pink as a test food. The chewing gum is placed on the tongue, and the subject is asked to chew it for 20 chewing cycles on his/ her preferred chewing side. It is then removed from the oral cavity and pressed in clear plastic film to a thickness of one mm. Both sides of the rolled-out chewing gum are then digitized using a flatbed scanner, and the two resulting images are copied into an image template of a specified size and number of pixels. The software Viewgum© [74] can be used to determine the variance of the color tones; this can be used to determine chewing efficiency. The color variance (Variance of Hue, VOH) shows a strong association with the number of chewing cycles and can be described by a logarithmic to linear curve, depending on the sample material. Hence, samples with a low color mixing degree show a high variance in the color distribution and indicate a poor chewing function [75]. Furthermore, van der Bilt et al. demonstrated that these two-colour mixing-ability tests might be more suitable for assessing chewing function in individuals with impaired mastication [70,75,76,77,78].

**Figure 1:**
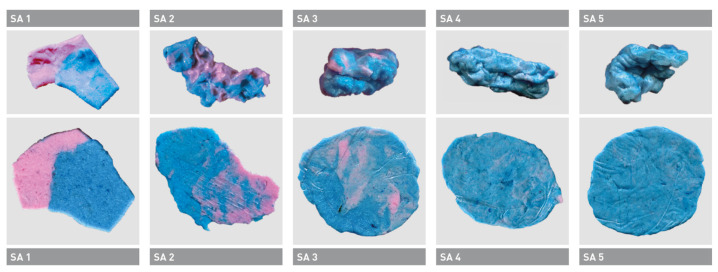
The Subjective Assessment Scale (SA) allows for the quick and simple evaluation of chewing efficiency by judging color mixture and bolus formation [63]. If the patient shows a degree of mixing of SA 1 or SA 2, it can be assumed that she/he has difficulty enjoying normal meals.

The group of Kaya et al. observed a correlation between a bolus kneading test based on the analysis of VOH and D50 (chewing efficiency) [76]. Both methods were able to differentiate masticatory performance differences. Nonetheless, the two-color chewing gum mixing ability test can still be considered reliable for assessing masticatory performance and chewing performance, especially in non-clinical settings for individuals with dysphagia or reduced chewing function. A simplified version of this color mixing test is also suitable for use in a dental practice, hospital or nursing home. For this, the bolus taken from the oral cavity is first evaluated visually using a scale and provides quick and simple information about the individual chewing efficiency (Figure 1).

If the patient shows a degree of mixing of 1 or 2, it can be assumed that he or she has difficulty enjoying normal meals. For example, if no material at all is available for testing the chewing function when a patient is admitted to the geriatric hospital, simply biting the examiner’s finger [79] or chewing a carrot on a trial basis [80] can provide an initial indication of whether the patient can be served pureed or normal meals.

**Table 1:** Recommended methods of assessment of chewing efficiency/performance, i.e., objective clinical assessment for frail older adults.

Test	Methodology	Functioning
Color mixing-ability test	Two-colored chewing gum	Might contain sugar, older adults might not be familiar with chewing gum, easy to control bolus, easy to evaluate, easy to evaluate (scale) [14,81].
Color mixing-ability test	Two-colored wax	Older adults might not be comfortable with chewing on wax, easy to control bolus, easy to evaluate, easy to evaluate (scale) [82].
Glucosensor © (GC)	Glucose extraction from gum jelly	Needs specialized equipment and specimens [83].
Carrot-test	Carrot slices	Always available, hardness might be difficult to control, easy to evaluate (scale) [80].
Bite force	Force gauge	Force gauges for bite force are often not available. Bite force, however, is a good predictor of chewing function [47].
Occlusal contacts in the four supporting zones (Eichner Classification)	Needs light and a good overview	Easiest way to extrapolate on chewing function. Main predictor of chewing function [8].

## 6. Chewing Ability

Considering the limitations of solely assessing chewing function with clinical tests, the chewing ability will be assessed with qualitative semi-structured interviews or validated questionnaires, such as the temporomandibular joint disability index (TDI), and the eating Related Quality of Life. However, in a recent study, there were no conclusive significant correlations between the subdomains of chewing ability or the nutritional variables. This could be due to the lack of standardized and validated methods for assessing masticatory ability for various cultural or geographical backgrounds. Although there is no widely accepted questionnaire, some instruments comprise questions that relate to chewing ability and some to specific compensatory mechanisms if chewing is impaired, as shown in Table 2.

## 7. Improvement the Masticatory Performance in Frail Older People

When considering different treatment modalities in the rehabilitation of edentulous individuals, Muller et al. [57] described the masticatory performance with two implant overdentures (IOD) in patients depending on their ADL (activities of daily living). The intervention group received two interforaminal short implants (*n* = 16), while the control group (*n* = 18) obtained conventional relines. The results showed that IODs were more stable and resulted in significantly higher denture satisfaction and OHRQoL compared to the control group. The study also showed an improvement in maximum voluntary bite force and masseter muscle thickness in the intervention group, indicating that IODs may benefit edentulous patients who require assistance even late in life [57].

In view of the different numbers and types of reestablishments of masticatory units, McKenna G et al. [88] conducted a randomized controlled trial in 2014, comparing removable dental partial prostheses to shortened dental arches restored with bridgework. Both groups showed significant improvement in masticatory performance (*p* < 0.0001) with no significant difference between the groups (*p* = 0.1689); however, significantly higher OHRQoL and reduced caries incidence in the group with fixed dental prostheses [88].

Several studies have examined various factors related to oral health, medical conditions, and nutrition that may affect masticatory function. In a 10-year longitudinal survey by the group of Sato et al. with the inclusion of 349 older adults, they found that occlusal support did not have a significant impact on masticatory ability. However, the number of food items that could be chewed had significantly decreased in subjects who remained in Zone A (subjects’ largest number of eatable items), suggesting that other factors beyond occlusal support (number of occluding pairs) may play a role in masticatory ability in very old individuals [62]. Adherence to a familiar Mediterranean diet in a sample of older Greek adults was discussed by the group of Bousiou et al. [89]. They detected that lower masticatory performance, higher BMI, smoking, and a larger number of drugs per day negatively affected adherence to the Mediterranean diet in older adults. Nevertheless, increased masticatory performance was an independent predictor of better adherence to the Mediterranean diet [89].

Based on the findings of animal and human experimental studies investigating the interplay of mastication, nutrition, cognition, and activities of daily living. Weijenberg et al. (2011) suggested a causal relationship between mastication and cognition [51]. Even though the healthy brain has an amazing capacity to adapt to changes and create new neural pathways, which is called neuroplasticity, when it comes to prosthodontic rehabilitation and the loss of periodontal receptors and neural pathways, the adaptive capacities of older patients may be limited. Additionally, there are currently no reliable predictors for the success of treatment in these cases. This highlights the importance of carefully considering the potential impact of invasive treatment concepts on the functional and cognitive abilities of aged patients.

A valuable alternative could be the alteration of the preexisting dental prosthesis to optimize masticatory function and simultaneously limit the adaption process needed [90,91]. A different option for complete dentures and IODs involves creating a duplicate prosthesis. This approach allows for the preservation of various aspects of the existing prosthesis, such as vertical dimension and aesthetics, while also fabricating a new prosthesis that offers improved hygiene [92].

The number and time of clinical visits during the fabrication of a new prosthesis should be limited and adjusted to patients’ medical conditions and, furthermore, be as atraumatic as possible. Different treatment modalities were proposed to limit the number of dental visits, i.e., the acquisition of the definitive impression and jaw relation in one session [93,94]. Nevertheless, it is crucial to evaluate each patient’s unique needs and ensure that the post-treatment care is designed to be easily manageable, with a focus on facilitation, especially in terms of the deconstructability and maintenance of denture and oral hygiene [3].

In terms of medical conditions, amyotrophic lateral sclerosis (ALS) is a progressive neurodegenerative disease that affects motor neurons. As the disease progresses, it leads to muscle weakness and atrophy, which can affect the ability to speak, swallow and breathe. Schimmel et al. [95] conducted a matched case-control study to investigate the oral function of ALS patients. The study involved 26 ALS patients and 26 matched controls, and the results showed that ALS patients had significantly lower chewing performance, lip force, tongue force, saliva weight, and fat-free mass index compared to controls. ALS patients also had a higher EAT-10 score. In addition, low chewing performance in ALS patients was found to be correlated with low bite and tongue force. Chewing performance, fat-free mass index, and saliva weight were identified as the most important discriminant parameters between the two groups [95].

In a longitudinal study with stroke patients, the group by Schimmel et al. evaluated chewing performance, lip and bite force, and masseter muscle thickness compared to controls over a 2-year period. Results showed impaired chewing efficiency and lower lip forces in stroke patients with no significant improvement over time. Bite forces were not different between sides, but hand-grip strength was significantly impaired and did not improve. Impaired chewing efficiency and reduced lip force are quantifiable symptoms in stroke patients that may require oro-facial rehabilitation [63].

The Japanese Society of Gerodontology proposes diagnostic criteria and management strategies to reduce the risk of oral dysfunction among older people, defining it as a presentation of seven oral signs or symptoms and recommending more evidence from clinical studies to clarify their diagnostic criteria and management strategies. Clinical signs include poor oral hygiene (total number of microorganisms [CFU/mL is 106.5 or more]), oral dryness (moisture checker < 27.0), reduced occlusal force (<200 N), decreased tongue-lip motor function (the number of any counts of pa/ta/or/ka/ produced per second is less than 6), decreased tongue pressure (maximum tongue pressure is less than 30 kPa), decreased masticatory function (the glucose concentration obtained by chewing gelatin gummies is less than 100 mg/dL) and deterioration of swallowing function (the total score of EAT-10 is 3 or higher) [10].

Schimmel et al. propose a conceptual model of oro-facial health that emphasizes the relationship between oro-facial function and an individual’s ability to lead an independent life until death. According to this model, a well-functioning oro-facial system is characterized by the absence of positive coping of physical and mental disease, pain and negative environmental and social factors. Conversely, oral hypofunction occurs due to physiological aging, comorbid medical conditions, and a lack of reliable assessment tools to distinguish between states of optimal oro-facial fitness and hypofunction [24].

## 8. Conclusions

- To fully encompass oral frailty, oro-facial hypofunction, or oro-facial fitness, dental patient reported outcomes (dPROs) should be included.
- Currently, there are few evidence-based rehabilitation approaches apart from prosthodontics to ameliorate oro-facial hypofunction.
- Older adults may have decreased neuroplasticity, which may hinder the effectiveness of such interventions, thus necessitating functional training and nutritional counseling to complement these strategies.
- The concept of oral frailty, oro-facial hypofunction or oro-facial fitness should involve dental patient reported outcomes (dPROs).
- Reduced neuro-plastic capacity in old individuals might preclude a positive outcome of these strategies that might need to be accompanied by functional training and nutritional counseling.

## Figures and Tables

**Table 2:** Examples of methods that relate to chewing ability, i.e., subjective evaluation of chewing.

Index	Methodology
TMJ Disability Index (TDI) [84]	Various items relate to difficulties in chewing certain examples of food items with varying consistencies. Hence, the TDI might be applicable to older adults with various cultural backgrounds.
Open or semi-structured interviews [85]	Individual evaluation of oral health and function, possible compensatory mechanisms, eating habits, and further adaptation processes. i.e., self-reported chewing difficulties, food avoidance,
Eating Related Quality of Life ERQoL [85,86]	Enjoyment of eating and social and emotional issues around eating, eating comfort
Denture satisfaction index [87]	VAS-scale-based instrument with certain items relating to chewing ability

## Data Availability

The data that support the findings of this study are available from the corresponding author upon reasonable request.
